# Association of HLA-G 14bp INS/DEL Polymorphism with brain morphology in Schizophrenia

**DOI:** 10.1186/1755-8166-7-S1-P43

**Published:** 2014-01-21

**Authors:** Ashwini Rajasekaran, Venkataram Shivakumar, Deepthi Venugopal, Sunil V Kalmady, Anekal C Amaresha, Mahavir Agarwal, Janardhanan C Narayanaswamy, Ganesan Venkatasubramanian, Monojit Debnath

**Affiliations:** 1 Department of Human Genetics, NIMHANS, Hosur Road, Bangalore-560029, India; 2 Translational Psychiatry Laboratory, Department of Psychiatry, Neurobiology Research Centre, NIMHANS, Hosur Road, Bangalore-560029, India

**Keywords:** HLA-G, Schizophrenia, imaging, biomarker

## Background

Multiple lines of evidence have implicated dysregulated immune processes in the pathogenesis of schizophrenia. Being an important immuno-modulatory molecule, Human Leukocyte Antigen (HLA)-G plays a pivotal role in successful pregnancy. Altered expression of HLA-G due to environmental and genetic variations not only lead to pregnancy complications but also a range of immunopathologies, some of which are being considered to confer risk for schizophrenia. One of the polymorphic marker, 14bp insertion/deletion (INDEL) located within the 3´UTR region of the HLA-G locus in Chr.6p21.3 is associated with HLA-G expression and function. The current study is aimed at analysing the role of 14bp polymorphism and the impact of feto-maternal compatibility/incompatibility at this locus on the risk of schizophrenia. In addition, the effect of 14bp INDEL on brain structure alterations in schizophrenia patients was also investigated.

## Methods

A total of 151 (male-75 and female-76) schizophrenia patients and 113 (male-68 and female-45) ethnicity matched healthy controls (HC) were considered. In addition, mothers of 64 schizophrenia patients were also recruited. Genotyping of 14bp INDEL was determined by PCR. Structural brain images were acquired using a 3-Tesla MRI in 108 HC and 76 schizophrenia patients. Voxel-Based Morphometry toolbox in SPM8 was utilized for brain imaging analysis using bilateral masks of the following brain regions: dorsolateral prefrontal cortex (DLPFC), hippocampus, parahippocampal gyrus (PHG) and posterior cingulate gyrus (PCG) [uncorrected p<0.01; small-volume-correction for the respective mask (family-wise-error) p<0.05; 10-voxel threshold].

## Results

There were no significant allele and genotype differences between patients and controls. However, a significant increase of heterozygous (+14bp/-14bp) genotype was observed in the female patients (p≤0.05). Interestingly, mother and the female patients also shared increased +14bp/-14bp compatibility. Imaging analysis indicated that patients exhibiting +14bp/-14bp genotypes had significantly deficient volume in the right hippocampus [30, -13, -12] and right PHG [41, -38, -11].

## Conclusion

Our results demonstrate a possible role of HLA-G polymorphism and feto-maternal matching of HLA-G in conferring the risk of schizophrenia. Importantly, this genetic variant also influences brain morphometric measures. Taken together, these findings suggest HLA-G could be an important biomarker for schizophrenia.

